# restfulSE: A semantically rich interface for cloud-scale genomics with Bioconductor

**DOI:** 10.12688/f1000research.17518.1

**Published:** 2019-01-07

**Authors:** Shweta Gopaulakrishnan, Samuela Pollack, BJ Stubbs, Hervé Pagès, John Readey, Sean Davis, Levi Waldron, Martin Morgan, Vincent Carey

**Affiliations:** 1 Channing Division of Network Medicine, Harvard Medical School, Boston, Massachusetts, 02115, USA; 2 Biostatistics and Computational Biology, Dana-Farber Cancer Institute, Boston, MA, 02115, USA; 3 Fred Hutchinson Cancer Research Center, Seattle, Washington, 98109, USA; 4 Tools and Cloud Technology, HDF Group, Seattle, WA, 98109, USA; 5 Center for Cancer Research, National Cancer Institute, USA, Bethesda, Maryland, 20892, USA; 6 Epidemiology and Biostatistics, CUNY School of Public Health, New York, New York, 10027, USA; 7 Biostatistics and Bioinformatics, Roswell Park Cancer Institute, Buffalo, New York, 14203, USA

**Keywords:** Bioinformatics, REST APIs, HDF5, BigQuery, Bioconductor

## Abstract

Bioconductor's SummarizedExperiment class unites numerical assay quantifications with sample- and experiment-level metadata.  SummarizedExperiment is the standard Bioconductor class for assays that produce matrix-like data, used by over 200 packages.  We describe the restfulSE package, a deployment of  this data model that supports remote storage.  We illustrate use of SummarizedExperiment with remote HDF5 and Google BigQuery back ends, with two applications in cancer genomics.  Our intent is to allow the use of familiar and semantically meaningful programmatic idioms to query genomic data, while abstracting the remote interface from end users and developers.

## Introduction

Analyses of multiomic archives like The Cancer Genome Atlas (TCGA) and single-cell transcriptomic experiments such as the 10x 1.3 million mouse neuron dataset typically begin with downloads of large files and conversion of file contents into formats based on local preferences. In this paper we consider how targeted queries of large remote genomic data resources can be conducted using methods available for Bioconductor’s *SummarizedExperiment* class. For large data archives that have been centralized in cloud storage, use of this approach can help diminish effort required to manage local storage, and can facilitate interactive analysis of data subsets in familiar programming idioms, without downloading entire datasets. Clients for HDF5 or Google BigQuery are available in numerous languages; our Bioconductor interface permits access to remote archives of genomic data with familiar and semantically meaningful programmatic idioms, while abstracting the remote interface from end users and developers.

## Methods: Data structures and remote back ends

### The SummarizedExperiment class and related methods

Let *Q* denote a matrix of quantifications arising from a genome scale assay with *G* assay features measured on *N* experimental samples. The elements of *Q* are the numbers *q _ij_*, *i* = 1, … , *G*, *j* = 1, …, *N*. Bioconductor’s SummarizedExperiment structure manages feature quantifications with associated metadata about assay features and samples.

**Figure 1.**
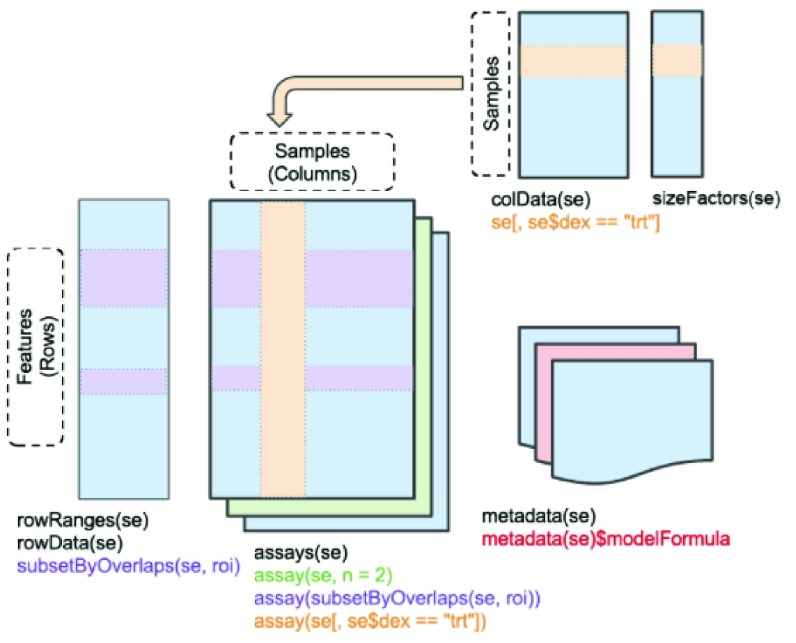
Schematic of SummarizedExperiment class structure. Colored regions of panels within the schematic are linked with command examples in colored text beneath the panels. For example, the purple command subsetByOverlaps(se, roi) would produce a restricted RangedSummarizedExperiment instance with features limited to those colored purple. The sizeFactors component is specific to a subclass for single cell data.

In the 10x mouse neuron dataset, *G* = 27998 and *N* = 1.3 million. Each of the *G* features is a gene, and it is useful to have handy a number of feature annotations like gene name, location, functional role; suppose each gene has *F* such features recorded. When these quantifications and associated annotations are managed in a Bioconductor SummarizedExperiment X, the matrix *Q* is programmatically bound to a *G* × *F* table of feature-level metadata accessible by the rowData method, and to an *N × R* table of sample-level metadata accessible by colData, where *R* denotes the number of sample-level metadata features recorded (Huber *et al.* ^1^). See Figure 1.

In the context of R programming, let K denote a vector of feature identifiers, S denote a vector of sample identifiers. The standard subsetting idiom X[K,S] expresses filtering of the all the information in *Q* and the associated metadata to features K and samples S. A GRanges instance (Lawrence *et al.* ^2^) defining genomic coordinates for features may be bound to X, facilitating queries defined by genomic location (using, for example, subsetByOverlaps) to isolate features coincident with or near the elements of a set of query genomic ranges (eg., binding peaks). This outline of genomic data representation and analysis is characteristic of Bioconductor.

### Examples of remote back ends

***Google BigQuery.*** The Institute for Systems Biology Cancer Genomics Cloud project (ISB-CGC) (ISB ^3^) uses Google BigQuery to provide access to various public cancer genomics resources including TCGA and the PanCancer Atlas (Hoadley *et al.* ^4^). The pancan_SE function of *restfulSE* constructs queries that derive SummarizedExperiment instances using quantifications and annotations for PanCancer atlas experiments managed in BigQuery tables.

***HDF Scalable Data Service (HSDS)*.** An AWS S3-based distributed data object model for HDF5 datasets, including a RESTful API to structure, populate, and query HDF5 archives, has been implemented by the HDF Group. A number of datasets of interest in bioinformatics are served through HDF Kita Lab in the /shared/bioconductor folder.

### Lazy data retrieval via DelayedArray

The *restfulSE* package provides interfaces to BigQuery and HSDS so that the numerical content housed in these services satisfies the API of the Bioconductor *DelayedArray* (Pagès and Hickey ^5^). Any DelayedArray instance can serve as the assay component of a SummarizedExperiment instance. Thus the capacities of SummarizedExperiment to bind semantically rich metadata to genome-scale assays are extended implicitly to data resources for which no standards exist for associating substantive metadata.

In conjunction with the *rhdf5client* and *bigrquery* packages, *restfulSE* functions translate filtering and selection operations which are readily defined using rowData, rowRanges, colData into formal queries resolvable by the HDF5 and BigQuery services. Numerical results are transmitted from server to client only when needed.

## Results

The RESTful SummarizedExperiment representation allows complicated research queries to be obtained in a concise, fast, convenient and robust fashion, as illustrated by the following examples.

### Hybrid data/annotation strategy for integrative analysis

**Figure 2.**
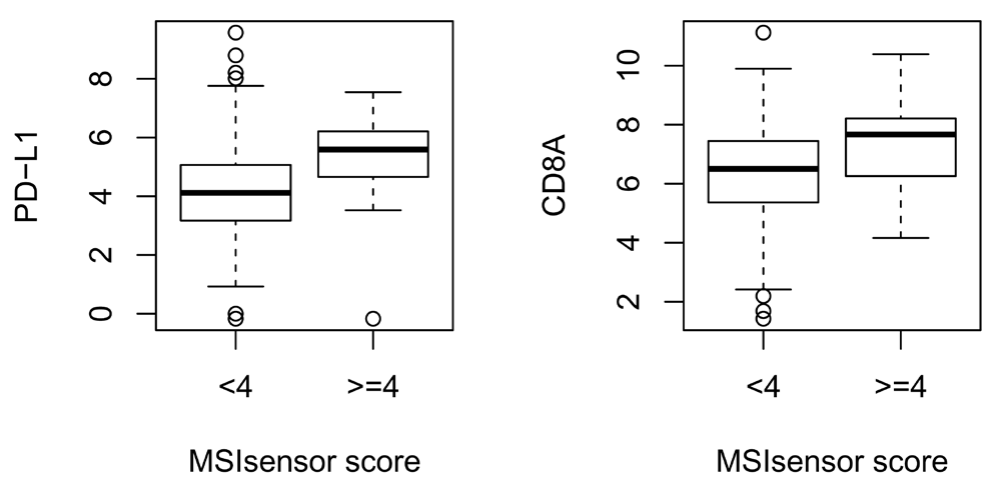
Association of MSI sensor scores with distributions of PDL-1 and CD8A in TCGA colorectal adenocarcinoma samples (COAD).

The following code chunk, which generates Figure 2, illustrates the use of the *restfulSE* protocol with the ISB-CGC BigQuery back end.

library(SummarizedExperiment)
library(BiocOncoTK)       # uses restfulSE for cancer bioinformatics
bq = pancan_BQ()          # need CGC_BILLING to authenticate
seCOAD = buildPancanSE(bq, acronym="COAD", assay="RNASeqv2")
seCOAD = bindMSI(seCOAD)  # update to include MSIsensor scores
par(mfrow=c(1,2))         # figure layout
amap = c("29126"="PD-L1", "925"="CD8A") # entrez:symbol mapping
bxs <- lapply( c("29126", "925"),       # for genes of interest
  function(x) boxplot(split(log2(as.numeric(assay( seCOAD[x,]))+1),
      seCOAD$msiTest >= 4), names = c("<4", ">=4"), ylab=amap[x],
      xlab="MSIsensor score")
  )

Our interest is in replicating part of Figure 5C of Bailey *et al.* ^6^. In that paper, it is shown that microsatellite instability (MSI) is associated with different expression signatures of immune cell infiltration for adenocarcinomas of colon (COAD) and stomach (STAD), and uterine corpus endometrial carcinoma (UCEC). The MSI scores developed using MSIsensor are found in Table S5 of Ding *et al.* ^7^. These scores are not available in BigQuery, but can be combined with the assay data using standard R programming, leading to a hybrid data/annotation strategy.

Functions in the *BiocOncoTK* package (Carey ^8^) build on *restfulSE* functionality to a) authenticate the user to the BigQuery platform, b) select a tumor type (COAD) and assay for *SummarizedExperiment* construction, c) bind Ding *et al*.’s MSI values as sample-level data variable msiTest, d) acquire and transform the PD-L1 and CD8A (Entrez IDs 29126 and 925) expression values, and e) form the stratified boxplot. The basic findings of Bailey *et al.* are replicated. Enhancement of the code to produce a display covering more genes and tumor types is demonstrated in the BiocOncoTK package vignette. Note that in this example, expression values are only downloaded for the genes requested, without altering the end user programming paradigm of working with a SummarizedExperiment instance.

### HDF Scalable Data Service

**Figure 3.**
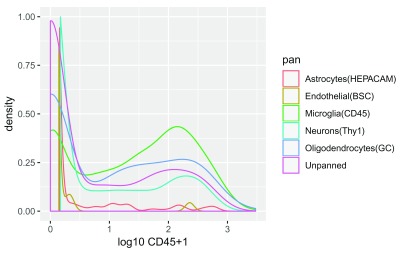
Density estimates for log10 CD45 expression in single-cell RNA-seq studies of glioblastoma.

Figure 3 demonstrates use of a RESTful SummarizedExperiment, with assay data provided in the object /shared/bioconductor/darmgcls.h5 at hsdshdflab.hdfgroup.org. Briefly, as a prelude to single-cell RNA-sequencing of glioblastoma (GBM) tumors from four patients, Darmanis *et al.* ^9^ used immunopanning to increase the proportion of non-neoplastic cells that constitute the “migrating front” of progression of glioblastoma. Antibody to CD45 was used to capture microglial cells. Figure 3 provides code to compare the distribution of CD45 expression among the classes of cells as labeled in the metadata of GSE84465, the NCBI GEO archive from which the quantifications were derived. In this example, data on one gene from all cells is retrieved when the statement defining vector vals is executed. The display can be recapitulated for other genes by substituting different symbols in the statement computing ind. The DelayedArray framework leveraged here enables basic computations of this kind without loading the entire matrix into memory.

library(rhdf5client)
library(SummarizedExperiment)
library(ggplot2)
cdar = BiocOncoTK::darmGBMcls
ind = match("PTPRC", rowData(cdar)$symbol)
var = gsub("selection: ", "",
       cdar$characteristics_ch1.8)
vals = log10(assay(cdar[ind,])+1)
ddd = data.frame(log10norm=vals, pan=var)
ggplot(ddd, aes(x=log10norm, colour=pan)) +
  geom_density() + ylim(0,1) +
  xlab("log10 CD45+1")

## Performance

We focus on pursuit of reliability, expressivity, and scalability using *restfulSE*.

**Reliability:** The *restfulSE*, *rhdf5client* and *BiocOncoTK* packages are accompanied by detailed unit tests that compare retrievals to known values. In the case of BigQuery table queries, the test suite composes random queries in both BigQuery SQL and in the SummarizedExperiment idiom. Results are checked for elementwise equality.

**Expressivity:** The code segments for Figure 2 and Figure 3 are complex but easy to break down. The joining and reshaping of pancan-atlas tables in BigQuery corresponding to the code in Figure 2 can be checked through the query history in the BigQuery interface. The acquisition of expression values employed five nested SELECT statements; the query for assay quantifications was 6000 characters in length. The R code is less than 500 characters including comments.

**Scalability.** BigQuery is intrinsically auto-scaling, but charges accrue with the amount of data scanned, so query design can have effects on throughput and cost. We rely on the *bigrquery* (Wickham ^10^) and *dbplyr* (Wickham and Ruiz ^11^) packages for efficient translation of R-oriented data manipulations to BigQuery SQL. Throughput with the HDF Scalable Data Service is dependent upon the configuration of the object server, the relationship of numerical data layout to prevalent access patterns, and the degree to which queries capitalize on API efficiencies like chunk-based retrieval. For both back ends, proper design and deployment of the querying client can lead to throughput that scale with client-side resources.

## Conclusions

Cloud-scale storage and retrieval strategies are of significant interest for genome science. The SummarizedExperiment class unifies assay data with substantive sample- and experiment-level metadata, and its API for managing and interrogating genome-scale experiment archives is used in numerous analytic packages. The *restfulSE* package exposes high-performance cloud-resident data stores to users and algorithms as SummarizedExperiments. Continued improvements in efficiency of representation and query resolution for assay data and metadata will help to achieve the potential of a federated data ecosystem for enhanced discovery in biology through interactive genome-scale analysis.

## Software availability

*restfulSE* package available from: https://bioconductor.org/packages/3.9/restfulSE Source code available from: https://github.com/shwetagopaul92/restfulSE Archived source code as at time of publication: DOI: 10.18129/B9.bioc.restfulSE ^12^ License: Artistic-2.0
