# Supplementary material for: restfulSE: A semantically rich interface for cloud-scale genomics with Bioconductor
Source: F1000Res. 2019 Jan 7;8:21. [Version 1] doi: 10.12688/f1000research.17518.1 (PMC6392152; doi:10.12688/f1000research.17518.1)
Supplement: Peer review history

# Peer review: Reviewer response for version 1

**DOI:** 10.5256/f1000research.19158.r42652

**Published:** 2019-02-25

**Authors:** Sheila Reynolds

**Affiliations:** 1 Institute for Systems Biology, Seattle, WA, USA

The restfulSE interface described in this article by Gopaulakrishnan et al. is a very useful extension to the SummarizedExperiment class which provides a convenient approach to storing and manipulating rectangular matrices of experimental results, along with associated meta-data. This new extension allows users to query remote data, eliminating the common “download” step that still precedes many large-scale analyses.

 As these datasets grow, and are more commonly made available in cloud-hosted technologies such as Google or AWS object stores or data warehouses such as Google BigQuery, tools that allow users to easily access and query these datasets become critical. The restfulSE interface permits targeted queries of such remote datasets.

 As background information, the article includes a nice summary of the SummarizedExperiment class and related methods, for researchers (such as this reviewer) who had not come across this package before. The authors go on to describe two separate remote back ends: one which accesses PanCancer Atlas TCGA, hosted in Google BigQuery by the ISB-CGC; and the other which access HDF5 data hosted in AWS S3. Both of these backends further make use of the DelayedArray package, which implements delayed or block-processing operations to facilitate working with large datasets that cannot be stored in-memory. This enables “lazy” data retrieval, with numerical results transmitted from server to client only when needed.

 The authors provide two concrete examples, illustrating the usage of both remote back ends. This reviewer ran into some issues trying to run these examples and reached out to the authors who provided additional information in video and Jupyter notebook form. Making additional tutorial resources available with this article will render this information useful and usable by a wider audience and is strongly encouraged.

I have read this submission. I believe that I have an appropriate level of expertise to confirm that it is of an acceptable scientific standard.

# Peer review: Reviewer response for version 1

**DOI:** 10.5256/f1000research.19158.r43093

**Published:** 2019-02-04

**Authors:** Dennis J. Hazelett

**Affiliations:** 1 The Center for Bioinformatics and Functional Genomics, Department of Biomedical Sciences, Cedars-Sinai Medical Center, Los Angeles, CA, USA

The restfulSE software package for bioconductor purports to extend a very useful data structure, the SummarizedExperiment to handle very large datasets wherein dynamic download of the full dataset is neither necessary nor practical. Therefore, Gopaulakrishnan et al. have created restfulSE to make this data structure interactive with remote databases on an as-needed basis. 

 This is a very useful idea from the Bioconductor core team, and likely to be impactful as datasets grow larger, cheaper to produce, and it becomes increasingly necessary for bioinformaticians to leverage available data against local experiments.

 The tool is technically sound, built on Google BigQuery and HDF5, and the paper is well written and clear. The manuscript includes code examples making it simple to get a quick start and see how the software works.

I have read this submission. I believe that I have an appropriate level of expertise to confirm that it is of an acceptable scientific standard.
